# Inhalation treatment of lung cancer: the influence of composition, size and shape of nanocarriers on their lung accumulation and retention

**DOI:** 10.7497/j.issn.2095-3941.2014.01.004

**Published:** 2014-03

**Authors:** Olga B. Garbuzenko, Gediminas Mainelis, Oleh Taratula, Tamara Minko

**Affiliations:** ^1^Department of Pharmaceutics, Ernest Mario School of Pharmacy, Rutgers, The State University of New Jersey, Piscataway, NJ 08854, USA; ^2^Department of Environmental Sciences, Rutgers, The State University of New Jersey, New Brunswick, NJ 08901, USA; ^3^Environmental and Occupational Health Sciences Institute, Piscataway, NJ 08854, USA; ^4^Department of Pharmaceutical Sciences, Oregon State University, Corvallis 97331, USA; ^5^Rutgers Cancer Institute of New Jersey, New Brunswick, NJ 08903, USA

**Keywords:** Nanoparticles, drug delivery systems, intravenous administration, lung neoplasms, inhalation administration

## Abstract

**Objective:**

Various nanoparticles have been designed and tested in order to select optimal carriers for the inhalation delivery of anticancer drugs to the lungs.

**Methods:**

The following nanocarriers were studied: micelles, liposomes, mesoporous silica nanoparticles (MSNs), poly propyleneimine (PPI) dendrimer-siRNA complexes nanoparticles, quantum dots (QDs), and poly (ethylene glycol) polymers. All particles were characterized using the following methods: dynamic light scattering, zeta potential, atomic force microscopy, *in vitro* cyto- and genotoxicity. *In vivo* organ distribution of all nanoparticles, retention in the lungs, and anticancer effects of liposomes loaded with doxorubicin were examined in nude mice after the pulmonary or intravenous delivery.

**Results:**

Significant differences in lung uptake were found after the inhalation delivery of lipid-based and non-lipid-based nanoparticles. The accumulation of liposomes and micelles in lungs remained relatively high even 24 h after inhalation when compared with MSNs, QDs, and PPI dendrimers. There were notable differences between nanoparticle accumulation in the lungs and other organs 1 and 3 h after inhalation or intravenous administrations, but 24 h after intravenous injection all nanoparticles were mainly accumulated in the liver, kidneys, and spleen. Inhalation delivery of doxorubicin by liposomes significantly enhanced its anticancer effect and prevented severe adverse side effects of the treatment in mice bearing the orthotopic model of lung cancer.

**Conclusion:**

The results of the study demonstrate that lipid-based nanocarriers had considerably higher accumulation and longer retention time in the lungs when compared with non-lipid-based carriers after the inhalation delivery. These particles are most suitable for effective inhalation treatment of lung cancer.

## Introduction

Inhalation delivery of drugs for the treatment of different lung diseases has attracted considerable interest from scientists in different research areas as well as clinicians^1^^-^^4^. The advantages of the lungs as a site of drug application are their large surface area, thin alveolar epithelium, easily permeable membrane and extensive vasculature, which allow substantial and rapid absorption of soluble and permeable active substances^5^. Therefore, drugs can be absorbed in the lungs to a relatively high extent after inhalation delivery.

It has been already reported that the local respiratory disorders and some systemic diseases can be successfully treated by delivering the drugs via pulmonary route. This includes the topical treatment of asthma, local infectious diseases, pulmonary hypertension, fibrosis *etc*^3^^,^^6^^-^^9^. Due to the limitations associated with the conventional treatment of lung cancer, a growing attention has been given to the development of the pulmonary route of drug administration directly to lungs^10^^,^^11^. Because of the distribution of lung cancer, cytoreductive surgery is not very effective for this disease, and therefore chemotherapy and/or radiation are the treatments of choice. The use of high doses of anticancer drugs leads to the fast development of cellular resistance and produces toxic effects on the entire body^12^. Local delivery of anticancer drugs via inhalation can increase their accumulation in lung tumor cells and reduce adverse side effects on healthy organs by limiting drug concentration in the blood^10^^,^^11^.

Drug formulation plays an important role in producing an effective inhalable medication. The following requirements have to be applied: (1) the formulation has to be pharmacologically active; (2) the formulation has to be delivered to the appropriate site of action; (3) the formulation has to remain in the lungs until the desired pharmacological effect occurs. Thus, it is necessary to develop a formulation that is capable of remaining in the lungs for the needed length of time while avoiding the clearance mechanisms in the lungs.

Nanocarriers for pulmonary application have been a popular topic within the last two decades. Different nanocarriers have been investigated for the pulmonary delivery including liposomes, polymeric micelles, dendrimers, mezoporous silica nanoparticles, nanostructured lipid carriers, *etc*^11^^,^^13^^-^^17^. The diversity of shapes, sizes, and components in various nanocarriers allows for selecting the best suitable delivery vehicle for a specific application. For example, liposomes, small lipid vesicles with a composition close to lung surfactant, have been successfully used for the delivery of different therapeutic agents to the lungs^10^^,^^18^^-^^20^. Micelles, representing spherical or globular structures that form when constituent molecules with a hydrophobic end clump to form the central core of the sphere in a liquid environment are useful for delivery of water-insoluble drugs carried in the hydrophobic central core^21^^,^^22^. Dendrimers, unimolecular, monodisperse, nanostructures, around 20 nm in size, with a well-defined, regularly branched symmetrical structure and a high density of functional end groups at their periphery, have been used in drug delivery studies. These nanocarriers are typically prepared using one or more of the following polymers: polyamidoamine (PAMAM), melamine, poly l-glutamic acid (PG), polyethyleneimine (PEI), polypropyleneimine (PPI), and polyethylene glycol (PEG)^23^^-^^27^. Mesoporous silica nanoparticles (MSNs) could also serve as suitable candidates for encapsulation and release of a variety of active pharmaceuticals due to their unique characteristics including a large surface area and high pore volume, as well as uniform porosity^16^^,^^28^^,^^29^.

For the last decade, our research group focused on the design, development, and application of different nanoformulations for the inhalation delivery of drugs, antisense oligonucleotides and siRNA for efficient cancer treatment in general, and lung cancer therapy in particular^10^^,^^11^^,^^16^^,^^17^^,^^30^. We concluded that the use of an appropriate nanocarrier for lung cancer treatment might help to extend drug retention time in the lungs, which in turn could substantially enhance the efficacy of the treatment. In addition to the type and characteristics of a nanocarrier, the route of the drug delivery to the targeted tissues could play a critical role in the treatment success. Hence, for the lung cancer therapy, we proposed a local pulmonary administration of the therapeutic nanocarriers by inhalation in order to simultaneously enhance the drug concentration in the lungs and decrease adverse side effects on other organs^11^^,^^16^^,^^17^. The present study is a logical continuation of our previous work and is aimed at *in vivo* analysis and comparison of different nanocarriers for their ability to penetrate the lung tissue and remain there for a sufficient time period after inhalation or intravenous delivery.

## Materials and methods

### Nanocarriers

**Figure 1:**
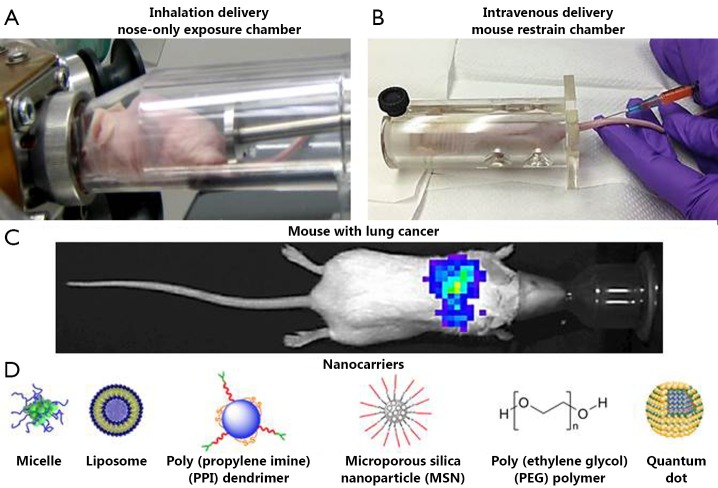
Experimental setup. (A) Nose-only exposure chamber for the inhalation delivery of drugs; (B) Mouse restrain chamber for the intravenous delivery of drugs; (C) Representative bioluminescent image of a mouse with orthotopic model of lung cancer (four weeks after instillation of cancer cells). Human A549 lung cancer cells transfected with luciferase were intratracheally injected into the lungs of nude mice. Intensity of bioluminescence is expressed by different colors, with blue reflecting the lowest intensity and green-yellow indicating the highest intensity; (D) Schematic representation of different nanoparticles used in the present study to deliver the therapeutics.

Different nanocarriers (**Figure 1**) were designed, prepared, and characterized using the procedures previously developed in our lab^31^. PEG polymers (2, 10 and 20 kDa) were purchased from Rapp Polymere GmbH (Tubingen, Germany). Neutral PEGylated liposomes were formulated from egg phosphatidylcholin: cholesterol, 1,2,-distearoyl-sn-glycero-3-phosphoethanolamine-N-aminopolyethelenglycol (DSPE-PEG) (Avanti Polar Lipids, Alabaster, AL, USA) in mole ratio 51^:^44^:^5, respectively, using the ethanol injection method with final lipid concentration 20 mM and loaded with doxorubicin (DOX) at 37 °C using two buffers with different pH for internal and external liposomal areas^32^^,^^33^. DOX DSPE-PEG (2 kDa) micelles were prepared as follows. DSPE-PEG powder was dissolved in tert-butanol, lyophilized overnight followed by rehydration in 0.9% NaCl to a final concentration above the critical micelle concentration (20 mM)^32^. Mobile Crystaline Material-41 (MCM-41) type MSNs were synthesized using a surfactant-templated, base-catalyzed condensation method as previously reported^16^^,^^29^^,^^31^. PPI tetrahexacontaamine dendrimers were obtained from Sigma-Aldrich and modified with PEG and complexed with siRNA as previously described^23^^,^^27^^,^^31^. Carboxyl terminated QDs (QD-COOH) with an emission peak at 490 nm were purchased from eBioscience, Inc. (San Diego, CA, USA). The structure of these functionalized nanocrystals consists of a core particle that is composed of cadmium selenide (CdSe) surrounded by a zinc sulfide (ZnS) shell. QDs have a lipid-based coating containing attached PEG molecules that enables water solubility, and contain carboxyl groups available for conjugation of other components. These quantum dots were previously evaluated and used in our lab as imaging agents and drug carriers^34^.

Particle size was measured by dynamic light scattering using 90 Plus Particle Size Analyzer (Brookhaven Instruments Corp., New York, NY, USA). To characterize the surface charge of nanoparticles, zeta potential was measured on PALS Zeta Potential Analyzer (Brookhaven Instruments Corp., Holtsville, NY, USA). The samples of nanocarriers were imaged with a tapping mode atomic force microscope (Nanoscope III A, Veeco Digital Instruments, Chadds Ford, PA, USA) as previously described^10^. Cyto- and genotoxicity of the studied nanocarriers were evaluated using the *in vitro* modified 3-(4,5-dimethylthiazol-2-yl)-2,5-diphenyltetrazolium bromide (MTT) and micronucleus assays as previously described31. Briefly, to study genotoxicity, about 300,000 cells were cultured with the media in 25 cm^2^ flasks and held 24 h before treatment. They were then incubated with tested nanocarriers for 24 h. Negative control cells were incubated with fresh media, while positive control cells were treated with ethyl methanesulfonate (400 g/mL). After incubation, the cells were fixed in a cold solution of 100% methanol. The methanol was removed and the cells were washed with phosphate buffer and the cells’ nuclei were then stained with 600 nM of 4,6-diamidino-2-phenylindole (DAPI) for eight minutes. This solution was removed and all the flasks were washed with PBS containing 0.05% Tween 20 (Sigma Aldrich, St Louis, MO, USA). After staining, the formation of micronuclei was detected by a fluorescent microscope (Olympus, New York, NY, USA) and documented by counting the number of micronuclei per 1,000 cells. All nanocarriers were conjugated with the near infrared fluorescent dye Cy 5.5 for the *in vivo* imaging (GE Healthcare Bio-Sciences Corp., Piscataway, NJ, USA).

### Cell lines

The experiments were carried out on two types of cells. A549 human non-small-cell lung adenocarcinoma epithelial cells transfected with luciferase (Xenogen, Alameda, CA, USA) were used for creating an orthotopic lung cancer model in nude mice. CHO-K1 cells (hamster ovary) were purchased from ATCC (Manassas, VA, USA) and used for study of genotoxicity of nanocarriers. A549 cells were cultured in RPMI 1640 medium (Sigma, St Louis, MO, USA) supplemented with 20% fetal bovine serum (Fisher Chemicals, Fairlawn, NJ, USA). CHO-K1 cells were cultured in Dulbecco’s modified Egle's medium (GIBCO Inc., Cincinnati, OH, USA) supplemented with 10% fetal bovine serum (Fisher Chemicals, Fairlawn, NJ, USA). All cells were grown at 37 °C in a humidified atmosphere of 5% CO_2_ (v/v) in air. The experiments were performed on cells in the exponential growth phase.

### Orthotopic animal model of lung cancer

Athymic nu/nu (NCRNU-M, CrTac: NCr-Foxn1nu) 6-8 weeks old mice were obtained from Taconic (Hudson, NY, USA). All mice were maintained in micro-isolated cages under pathogen free conditions in the animal maintenance facilities of Rutgers, The State University of New Jersey. The research involving animals has been reviewed and approved by institutional ethical committee before conducting research. An orthotopic mouse model of human lung cancer previously developed in our laboratory^11^^,^^16^^,^^17^ was used in the present study. Briefly, A549 human lung adenocarcinoma epithelial cells (5×10^6^-8×10^6^) transfected with luciferase were re-suspended in 0.1 mL of RPMI medium containing 20% fetal bovine serum, mixed with 5 µL of EDTA, and then administered to the mouse lungs through a catheter. The development of tumor growth was monitored and tumor volume was calculated as previously described^11^. To visualize lung tumor (**Figure 1**) and analyze the distribution of nanocarriers, imaging procedures were performed using *in vivo* bioluminescent/fluorescent IVIS (Xenogen, Alameda, CA, USA) system under inhalation anesthesia with isoflurane at a concentration of 4% for induction of anesthesia and 1%-2% for its maintenance during the imaging process. The quantitative analysis of tumor size and volume using optical imaging system was performed as described previously^10^^,^^11^^,^^35^.

### Exposure system

**Figure 2:**
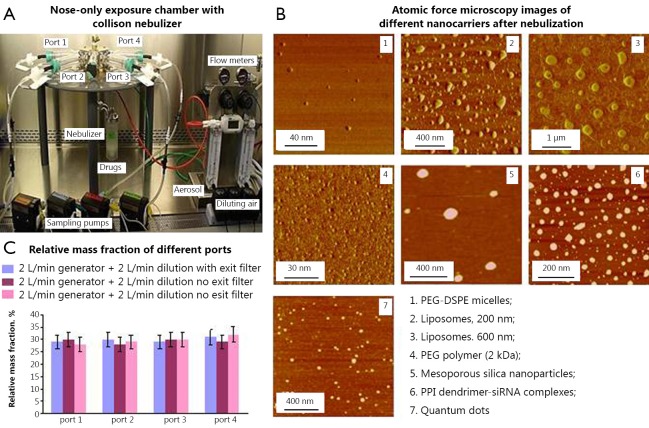
Characteristics on aerosolized nanocarriers. A, Collection of aerosolized nanoparticles from four ports of nose-only exposure chamber connected to a Collison nebulizer; B, Representative atomic force microscopy images of different nanocarriers after nebulization. Panoramic views were captured in phase contrast mode; C, Mass distribution of nanoparticles aerosolized using different regimens of nebulization between different ports.

A one-jet Collison nebulizer (BGI Inc., Waltham, MA, USA) operated at an aerosolization flow rate of 2 L/min using dry and purified air (Airgas East, Salem, NH, USA) was used to aerosolize particles while an additional air flow of 2-3 L/min was introduced to dilute and desiccate the resulting aerosol according to the previously described procedure^30^. Briefly, the Collison nebulizer was equipped with a precious fluid cup to minimize the amount of liquid needed for reliable aerosolization. Then, under a slight positive pressure, the entire aerosol flow of 4-5 L/min entered a mixing box of the 5-port exposure chamber (CH Technologies, Westwood, NJ, USA) and was distributed to each animal containment tube via round pipes (four out of five chambers were used in the experiments). Each containment tube was connected to the distribution chamber via a connector cone, which features a spout in its middle to deliver fresh aerosol to a test animal and round openings in its back for exhaled air. During the inhalation experiments, each tested animal was positioned in a containment tube so that the animal’s nose was at the spout, or ‘inhalation point’ (**Figure 1**). The animal was held in place by a plunger. The air exhaled by the test animal escapes the connector cone via openings in the cone’s back and was exhausted. In order to analyze the distribution of nanoparticles between the exposure chambers, green fluorescent polystyrene latex (PSL) particles (Duke Scientific, Palo Alto, CA, USA) of 0.2, 0.5 and 1.2 µm in size were used. PSL particles were aerosolized similarly to the nanocarriers and were collected from each chamber using sampling probes and filters connected to pumps (**Figure 2A**)^30^. The relative mass concentration of PSL particles collected on each filter was quantified by a fluorometer (FM109515, Sequoia-Turner Corp., Mountain View, CA, USA) using a previously described procedure^30^. The characterization experiments were performed with and without a filter attached to the exit port of the exposure system (configuration with no filter at the top of the system is shown in **Figure 2A**).

In order to analyze the effect of aerosolization on the stability and characteristics of nanocarriers as well as distribution of nanoparticle aerosols between different ports of the exposure system, we used the setup shown in **Figure 2A**. The system included the aerosol distribution chamber and animal containment tubes numbered from the port 1 (P1) through port 4 (P4), while the middle port was closed. In these experiments, the containment tubes were equipped with sampling probes connected via sampling filters with pumps. The inlet of each tube was positioned approximately where a mouse nose would be during actual exposure experiments. Samples of nanoparticles were collected on a filter from each port and analyzed to determine mass distribution uniformity across the sampling ports.

### Distribution of nanocarriers in different organs

The distribution of different nanocarriers was examined in nude mice after intravenous or inhalation administration as previously described^10^^,^^11^^,^^16^^,^^17^. Intravenously administered volume was 100 μL for each formulation. For inhalation administration, aerosolization of 100 µL of suspension resulted in inhaled volume of ~1.2 µg by each mouse according to our earlier estimates^30^. Each experimental group consisted of minimum 6 mice. Animals were anesthetized with isoflurane (1-2%, 100-200 mL/min) and euthanized, 1, 3 and 24 h after the treatment. Lungs, liver, spleen, and kidneys were excised, rinsed in saline, and fluorescence was registered by IVIS imaging system (Xenogen Corporation, Alameda, CA, USA). Images of each organ were scanned and total fluorescence intensity was calculated using the manufacturer software. The fluorescence was expressed in arbitrary units with one unit representing approximately 2×10^10^ photons/s/sr/cm^2^. The method allows a quantitative comparison of the concentration of the same fluorescent dye between different series of the experiments. The mass of all organs was measured and the fluorescence intensity was normalized by organ mass.

### Antitumor efficacy

In order to compare the antitumor efficacy of a selected nanocarrier containing the anticancer drug (600 nm liposomes loaded with DOX) after intravenous and inhalation delivery, apoptosis induction in different organs (the lungs with tumor, liver, kidney, spleen, heart and brain) was measured using Cell Death Plus ELISA kit (Hoffmann-La Roche). Four weeks after the instillation of tumor cells, mice were treated on days 0, 3, 7, 11, 14, 17, 21, and 24 with free DOX (intravenous injection) and liposomal DOX (intravenous and inhalation administrations). The dose of DOX in all formulations for intravenous administration was 2.5 mg/kg for the single injection. This dose corresponds to the maximum tolerated dose of free DOX injected intravenously^36^^,^^37^. For inhalation experiments we estimate that the dose was 14 µg/kg^30^.

### Statistical analysis

Data were analyzed using descriptive statistics, single-factor analysis of variance (ANOVA), and presented as mean values ± the standard deviation (SD) from 6 to 10 independent measurements. The comparison among groups was performed by the independent sample Student’s *t*-tests. The difference between variants is considered significant if *P*<0.05.

## Results

### Characterization of nanocarriers

The data summarized in **Table 1** show that the size of the studied particles varied from approximately 10 to 600 nm. Therefore, the used drug delivery carriers covered the entire size range designated for ‘nanocarriers’. The zeta potential data of different nanocarriers show that micelles and liposomes possessed a slightly negative charge. Our finding that zeta potential of lipid assemblies containing PEG-DSPE is negative is in agreement with the previously reported data^37^. It also supports the ‘hidden charge effect’ that was suggested for liposomes sterically stabilized through grafting of mPEG-DSPE by PEG moiety with molecular weight exceeding 0.75 kDa^38^. According to the conventional classification of nanocarriers, used liposomes and micelles can be considered “neutral.” The surface charges of all other particles were close to zero. Our previous investigations showed that all nanocarriers demonstrated no signs of cytotoxicity in concentrations used in the present study^31^. The genotoxicity data obtained in this study (**Table 1**) show that all used carriers did not induce additional micronuclei formation when compared with a spontaneous micronuclei formation in control (cells incubated with media) and can be considered as non-genotoxic.

**Table 1:** Size, zeta potential and genotoxicity of studied nanocarriers^†^

Nanocarrier	Size (nm)	Zeta potential (mV)	Genotoxicity (micronuclei per 1,000 cells)
DSPE-PEG micelles	16.5±4.0	–12.0±2.0	13.2±2.1
EPC-Chol-DSPE-PEG liposomes (200 nm)	200.0±25.1	–8.1±3.0	10.1±2.1
EPC-Chol-DSPE-PEG liposomes (600 nm)	600.0±40.2	–10.2±3.8	12.0±2.0
PEG polymer (2 kDa)	10.1±1.8	~0	8.0±2.3
PEG polymer (10 kDa)	12.0±1.5	~0	9.1±2.0
PEG polymer (20 kDa)	12.0±2.0	~0	10.2±3.0
QDs	33.3±4.2	–4.0±1.0	9.8±2.1
MSNs	160.0±10.5	~0	8.8±1.7
PPI dendrimer-siRNA complexes	200.0±20.6	~0	7.1±0.6
Media (negative control)	N/A	N/A	14.0±2.2
Ethyl methanesulfonate (positive control)	N/A	N/A	52.0±5.9*

^†^Mean ± SD are shown; *P<0.05 when compared with negative control.

The typical atomic force microscope (AFM) images of different nanoparticles collected after nebulization are presented in **Figure 2B**. The images revealed convex meniscus shaped particles that were uniformly distributed on the mica surface. No substantial presence of aggregates was observed as expected from physico-chemical properties of uniformly charged particles. The shape of nanoparticles was preserved during the process of nebulization. This is notable because during aerosolization by a Collison nebulizer 20 mL of suspension is re-circulated every six seconds. Analysis of size and surface charge of the nanoparticles also did not detect statistically significant changes in these properties before and after the nebulization process. **Figure 2C** shows the relative aerosol mass fraction across the four ports obtained with the fluorescently labeled particles. As can be seen, the particle mass distribution across the four used ports is rather uniform and no statistically significant differences among the ports were detected.

### Organ distribution of different nanocarriers

The distribution of all studied nanocarriers labeled with fluorescent dye Cy 5.5 in lungs, liver, kidneys and spleen was analyzed using IVIS imaging system within 1, 3, and 24 h after their intravenous or inhalation administrations. The total organ fluorescence was normalized with organ weight.

#### Lipid-based nanocarriers

**Figure 3:**
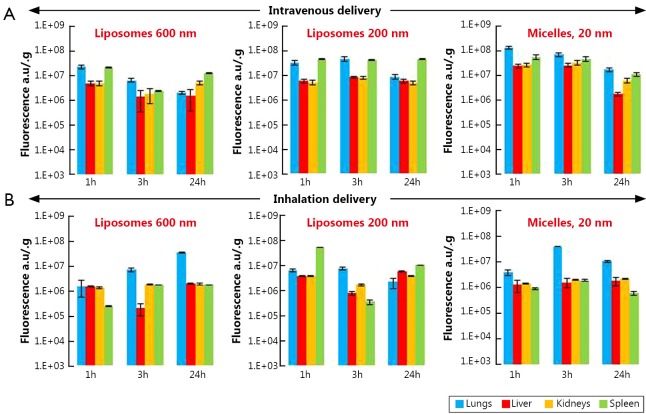
Organ distribution of liposomes (200 and 600 nm) and PEG-DSPE micelles in mice 1, 3 and 24 h after their intravenous or inhalation administration. All nanocarriers were labeled with near-infrared fluorescent Cy5.5 dye; total fluorescence in organs was measured using the IVIS Xenogen optical imaging system, and normalized per entire organ weight. A, Intravenous delivery; B, Inhalation delivery. Means ± SD are shown.

Our data showed that 1 h after intravenous injection of liposomes of different size (200 and 600 nm) and PEG-DSPE micelles (15 nm), they had almost identical distribution profile with preferential nanocarrier accumulation in the lungs and spleen (**Figure 3A**). As was expected, 24 h after intravenous injection the concentration of liposomes and micelles in the lungs decreased substantially compared with 1-hour period. The concentration of 200 and 600 nm liposomes in liver, kidneys, and spleen remained very similar at all investigated time points. On the other hand, nanocarrier distribution among different organs following inhalation delivery revealed higher non-uniformity in the distribution of nanoparticles (**Figure 3B**). Lung concentration of 600 nm liposomes at 3 and 24 h after inhalation was almost 4.5 and 23 times higher when compared with 1 h (please note a logarithmic scale of the ordinate in **Figure 3** and other distribution figures). At the same time, liver, kidney, and spleen exposure to these liposomes was lower than lung exposure at each studied time point and almost did not change with time. The distribution pattern of 200 nm liposomes in lungs and other organs at different time points after the inhalation administration was comparable to those after the intravenous administration with the peak concentration in the lungs at 3 h. Similar to 200 nm liposomes, the highest PEG-DSPE micelle concentration in the lungs was observed 3 h after its inhalation delivery followed by the concentration reduction at 24 h. However, in contrast to the intravenous administration profile, micelle concentration after their inhalation administration still remained higher when compared with 1 h after administration (**Figure 3B**). Overall, inhalation delivery of liposomes and micelles substantially reduced their concentration in non-target organs, such as liver, kidneys, and spleen, at all studied time points when compared with the intravenous administration.

#### PEG-polymers

**Figure 4:**
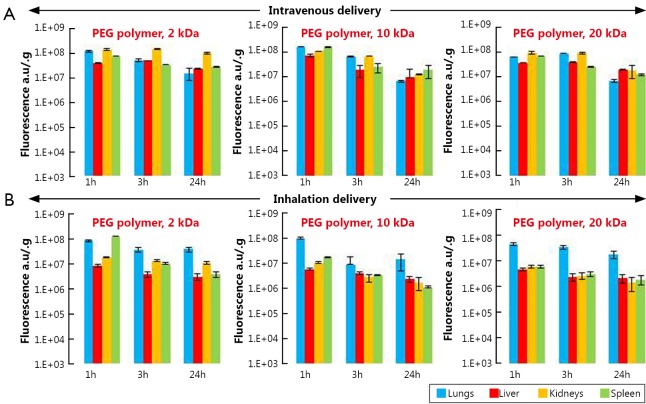
Organ distribution of 2, 10, and 20 kDa PEG polymers in mice 1, 3 and 24 h after their intravenous or inhalation administration. All polymers were labeled with near-infrared fluorescent Cy5.5 dye; total fluorescence in organs was measured using the IVIS Xenogen optical imaging system and normalized per entire organ weight. A, Intravenous delivery; B, Inhalation delivery. Means ± SD are shown.

Analysis of the accumulation of PEG polymers in organs after their intravenous delivery revealed that 2 and 10 kDa PEG polymers had very similar lung distribution pattern with the highest concentration at 1 h followed by the gradual reduction at later time points (**Figure 4A**). On the other hand, the highest concentration of 20 kDa PEG in the lungs was found 3 h after intravenous administration. The concentrations of 2 and 20 kDa PEG polymers in the kidneys were higher than those in the liver and spleen at all three time points. No significant differences were found between polymer concentrations in the lungs and other organs after their intravenous administration at all study time points. However, after inhalation administration, organ distribution profiles of all three polymers were substantially different from those after their intravenous administration (**Figure 4B**). At all studied time points, lung concentrations of 10 and 20 kDa PEG polymers were significantly higher when compared with their concentrations in liver, kidneys, and spleen. It was found that 2 kDa PEG polymer showed the same concentration profile as 10 and 20 kDa PEG polymers 3 and 24 h after inhalation, while 1 h after the inhalation administration the concentration of 2 kDa PEG polymer in lungs and spleen as almost identical. Notably, concentrations of all three polymers in liver and spleen were approximately two times lower after their inhalation delivery when compared with intravenous injection. The overall lung exposure calculated for all three polymers 24 h after their inhalation administration was 9.5 times higher than that in liver, 8.6 times higher than that in kidney, and 11.4 times higher than that in spleen while after intravenous delivery the overall lung concentration of polymers 24 h after their administration was almost three times lower than that in other studied organs.

#### Non-lipid nanocarriers

##### MSN

**Figure 5:**
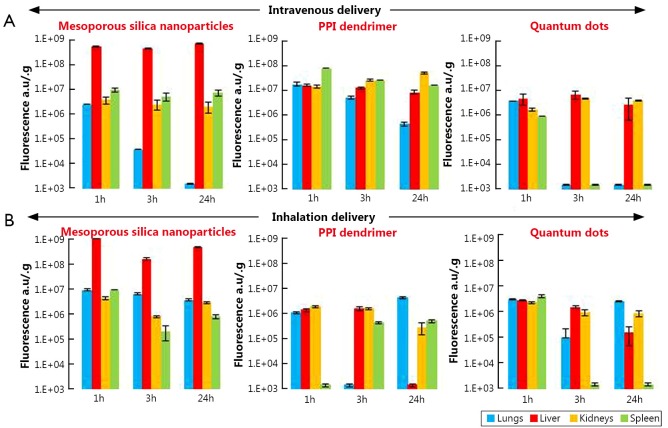
Organ distribution of MSNs, PPI dendrimer-siRNA complexes and QDs in mice 1, 3 and 24 h after their intravenous or inhalation administration. All nanocarriers were labeled with near-infrared fluorescent Cy5.5 dye; total fluorescence in organs was measured using the IVIS Xenogen optical imaging system and normalized per entire organ weight. A, Intravenous delivery; B, Inhalation delivery. Means ± SD are shown.

Analysis of organ distribution of MSN showed their preferential accumulation in the liver after either intravenous or inhalation administrations at all studied time points (**Figure 5**). The concentration of MSN in the lungs achieved its maximum value 1-hour after inhalation delivery followed by a decrease at 3 and 24 h. At the same time, only traces of MSN were detected in the lungs 24 h after their intravenous injection. The accumulation of MSN in the kidneys and spleen after inhalation showed a similar pattern to lung accumulation.

#### PPI dendrimer

The organ distribution pattern of PPI dendrimer after intravenous administration was similar to that of liposomes, with the highest lung concentration at 1 h and the lowest concentration at 24 h after injection (**Figure 3A** and **Figure 5A**). The concentrations of PPI dendrimers in the liver and kidneys were very similar to their concentrations in the lungs at 1 h and were higher than in the lungs 3 and 24 h after intravenous administration (**Figure 5A**). The organ distribution profile of PPI dendrimer after inhalation delivery was as follows: the highest concentration in the lungs and lowest concentrations in the liver, kidney and spleen was observed at 24 h when compared with 1 and 3 h time points (**Figure 5B**).

#### QDs

For intravenous administration, the presence of QDs in the lungs and spleen was observed only 1 h after their administration, but the liver and kidney concentration of QDs was almost identical at all study points (**Figure 5A**). However, after inhalation administration, the QDs concentration in the lungs at 24 h was approximately 2 and 1.5 times higher than those in liver and kidneys, respectively. No significant differences were observed in the lung, liver, kidney, and spleen concentrations of QDs 1 h after their inhalation delivery.

**Figure 6:**
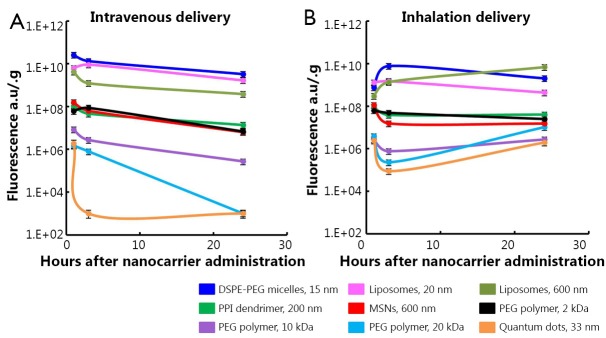
Accumulation of different nanocarriers in mouse lungs after intravenous and inhalation administration. A, Intravenous delivery; B, Inhalation delivery. Means are shown, means ± SD are shown.

The comparison of the accumulation and retention of different nanocarriers (**Figure 6**) showed that for most nanocarriers (except from MSN and 200 nm liposomes), their lung concentration retention time after inhalation delivery as longer when compared with that after intravenous injection.

### Antitumor efficacy

**Figure 7:**
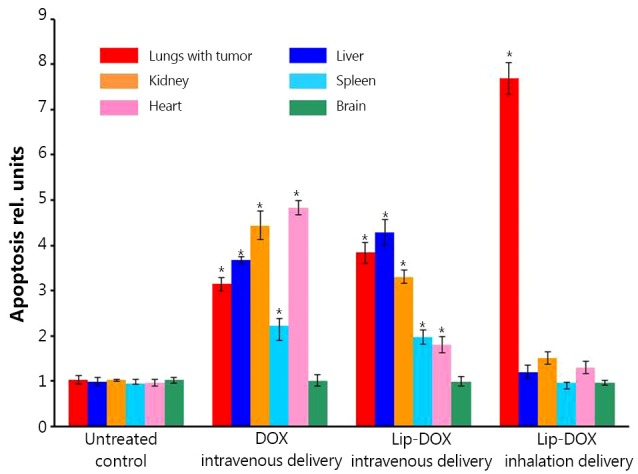
Apoptosis induction in the lungs with tumor and other organs in mice with lung cancer four weeks after beginning of treatment. Animals were treated two times per week with substances indicated. An orthotopic lung tumor model was created in nude mice by intratracheal instillation of A549 cells (non-small-cell lung carcinoma). Control mice received intravenous injections of saline. Means ± SD are shown. *P<0.05 when compared with untreated control.

Based on our organ distribution and lung retention data, the larger liposomes (600 nm) were selected as carriers for *in vivo* experiments. The analysis of apoptosis induction in the lungs with tumor and other organs after treatment of animals with lung tumor (**Figure 7**) showed distinct advantages of inhalation delivery of the anticancer drug. Inhalation delivery of liposomal DOX not only enhanced its antitumor effect but also limited adverse side effects of the treatment on healthy organs by preventing cell death induction.

## Discussion

Our previous data demonstrated that the composition, size, and shape of nanocarriers determine their body distribution, cyto- and genotoxicity, and consequently potential medical applications^11^^,^^16^^,^^17^^,^^26^^,^^31^^,^^34^^,^^35^. In addition, the route of administration also contributes to the overall distribution profile of nanoparticles and therefore influences their efficiency to treat diseases in the particular organs and ability to limit adverse side effects. In the present work we analyzed and compared in similar experimental conditions six different types of nanocarriers: polymeric micelles, liposomes, PEG polymers, PPI dendrimer, MSNs, and QDs for their ability to accumulate in the lungs and other organs after pulmonary and intravenous delivery. This study was also aimed at selecting a nanocarrier that could serve as the best delivery system for the lung cancer therapy. Finally, the anticancer effect of the most promising nanocarrier was confirmed in *in vivo* experiments on mice bearing the orthotopic lung cancer.

We found that both types of lipid based particles (micelles and liposomes) showed relatively high accumulation in the lungs especially at 1 and 3 h after intravenous administration followed by substantial decrease of their lung concentration at 24 h. This distribution pattern slightly depended on particle size. On the other hand, the high distribution volume of both liposomes and micelles was also found in other tissues (liver, kidney and spleen) after intravenous administration at all studied time points. Moreover, 24 h after injection the concentrations of 600 nm liposomes and 16 nm micelles in the kidney were notably higher than those in lungs. Very similar organ distribution profiles were found for all studied PEG polymers after their intravenous delivery to mice. Compared to PEG polymers, MSNs, PPI dendrimer, and QDs did not show elevated accumulation in the lungs when compared with other organs at any study points after their intravenous administration. Furthermore, their concentrations in the lungs were substantially lower at 24 h after intravenous administration compared to 1 and 3 h after administration.

Analysis of the organ distribution profiles after inhalation delivery showed that detectable concentrations of all nanocarriers were found in the lungs at all studied time points. However, PEGylated liposomes, PEG-DSPE micelles and PEG polymers showed a strongest tendency to be accumulated to the lungs and retain there at higher concentrations for the longer periods of time compared to MSNs, PPI dendrimer, and QD. These results are supported by the available literature reports that morphology, shape and size of nanoparticles can result circulation time in the body, as well as their interaction with different tissues *in vivo*^39^^,^^40^. Moreover, the present data clearly demonstrate that a route of administration is strongly associated with nanoparticles’ organ distribution and retention. When nanocarriers were administered by inhalation to mice, the extent of their accumulation and retention in the lungs did not depend as strongly on the nanocarrier type compared to intravenous injection. However, lipid-based particles (liposomes and micelles) exhibited a higher ability to accumulate in the lungs when compared with other studied particles. In order to confirm that such favorable organ distribution of lipid-based nanoparticles can result in high antitumor effect for the treatment of lung cancer, we studied the cell death induction in the lungs and other organs by DOX delivered by liposomes via inhalation and found that inhalation of liposomal DOX led to a significantly higher cell death induction in the lungs and substantially limited adverse side effects on healthy organs when compared with free or liposome-bound DOX delivered intravenously. Consequently, encapsulation of anticancer drugs in lipid-based nanocarriers and their delivery by inhalation enhanced the treatment efficiency of lung cancer and limited adverse side effects of the treatment.
